# Supplementary material for: Psychotropic deprescribing across different prescribing professions in New Mexico and Louisiana
Source: PLOS Ment Health. 2025 Dec 30;2(12):e0000408. doi: 10.1371/journal.pmen.0000408 (PMC12798385; doi:10.1371/journal.pmen.0000408)
Supplement: S1 Text — Table A: Covariate Balance Before Weighting. Table B: Provider Type by Year (Psychiatrist Cohort). Table C. Provider Type by Employment Status (Psychiatrist Cohort). Table D: Provider Type by Salary Type (Psychiatrist Cohort).Table E: Provider Type by Relationship to Employee (Psychiatrist Cohort). Table F: Provider Type by Rurality (Psychiatrist Cohort). Table G: Provider Type by Index Medication Type (Psychiatrist Cohort). Table H: Provider Type by Year (Primary Care Physician Cohort). Table I. Provider Type by Employment Status (Primary Care Physician Cohort). Table J: Provider Type by Salary Type (Primary Care Physician Cohort). Table K: Provider Type by Relationship to Employee (Primary Care Physician Cohort). Table L: Provider Type by Rurality (Primary Care Physician Cohort). Table M: Provider Type by Index Medication Type (Primary Care Physician Cohort). Fig A: Covariate Balance After Weighting (Psychiatrist Cohort). Fig B: Covariate Balance After Weighting (Primary Care Physician Cohort). (DOCX) [file pmen.0000408.s001.docx]

**S1 Text. Covariate Balance Before and After IPTW Weighting**

**Contents:**

Table A: Covariate Balance Before Weighting

Table B: Provider Type by Year (Psychiatrist Cohort)

Table C. Provider Type by Employment Status (Psychiatrist Cohort)

Table D: Provider Type by Salary Type (Psychiatrist Cohort)

Table E: Provider Type by Relationship to Employee (Psychiatrist Cohort)

Table F: Provider Type by Rurality (Psychiatrist Cohort)

Table G: Provider Type by Index Medication Type (Psychiatrist Cohort)

Table H: Provider Type by Year (Primary Care Physician Cohort)

Table I. Provider Type by Employment Status (Primary Care Physician Cohort)

Table J: Provider Type by Salary Type (Primary Care Physician Cohort)

Table K: Provider Type by Relationship to Employee (Primary Care Physician Cohort)

Table L: Provider Type by Rurality (Primary Care Physician Cohort)

Table M: Provider Type by Index Medication Type (Primary Care Physician Cohort)

Fig A: Covariate Balance After Weighting (Psychiatrist Cohort)

Fig B: Covariate Balance After Weighting (Primary Care Physician Cohort)

**Table A. COVARIATE BALANCE BEFORE WEIGHTING**

|  | **Psychologists vs Psychiatrists** | | | **Psychologists vs Primary Care Physicians** | | |
| --- | --- | --- | --- | --- | --- | --- |
|  | Prescribing Psychologists | Psychiatrists |  | Prescribing Psychologists | Primary Care Physicians |  |
|  | N (%) | N (%) | *p*-value | N (%) | N (%) | *p*-value |
| **Total N** | 2,755 | 10,793 |  | 2,345 | 103933 |  |
| **Predisposing** |  |  |  |  |  |  |
| Age at Service Date, M (SD) | 30.00 (16.03) | 31.59 (15.16) | <.0001 | 30.87 (16.03) | 36.74 (15.55) | <.0001 |
| Male | 1178 (42.76) | 4710 (43.64) | 0.4052 | 955 (40.72) | 40670 (39.13) | 0.1179 |
| **Enabling** |  |  |  |  |  |  |
| Employment Status |  |  | 0.0007 | 695 (29.64) | 28976 (27.88) | 0.2279 |
| Full-Time | 750 (27.22) | 3296 (30.54) |  | 10 (0.43) | 486 (0.47) |  |
| Part-Time | 12 (0.44) | 57 (0.53) |  | 18 (0.77) | 1007 (0.97) |  |
| Retired | 20 (0.73) | 126 (1.17) |  | 1622 (69.17) | 73464 (70.68) |  |
| Other/Unknown | 1973 (71.62) | 7314 (67.77) |  |  |  |  |
| Employment Type |  |  | 0.003 |  |  | 0.0043 |
| Salary | 289 (10.49) | 1288 (11.93) |  | 269 (11.47) | 10110 (9.73) |  |
| Hourly | 255 (9.26) | 1168 (10.82) |  | 226 (9.64) | 11395 (10.96) |  |
| Unknown | 2211 (80.25) | 8337 (77.24) |  | 1850 (78.89) | 82428 (79.31) |  |
| Relation to Employee |  |  | <.0001 |  |  | <.0001 |
| Employee | 1285 (46.64) | 5379 (49.84) |  | 1109 (47.29) | 60492 (58.2) |  |
| Spouse | 401 (14.56) | 1783 (16.52) |  | 373 (15.91) | 21786 (20.96) |  |
| Child/Other Dependent | 1069 (38.8) | 3631 (33.64) |  | 863 (36.8) | 21655 (20.84) |  |
| **Need** |  |  |  |  |  |  |
| Mental Health Diagnoses |  |  |  |  |  |  |
| Schizophrenia | 21 (0.76) | 179 (1.66) | 0.0005 | 10 (0.43) | 217 (0.21) | 0.024 |
| Bipolar Disorders | 95 (3.45) | 654 (6.06) | <.0001 | 43 (1.83) | 516 (0.5) | <.0001 |
| Depressive Disorders | 847 (30.74) | 3031 (28.08) | 0.0058 | 645 (27.51) | 7822 (7.53) | <.0001 |
| Anxiety Disorders | 945 (34.3) | 2746 (25.44) | <.0001 | 766 (32.67) | 10143 (9.76) | <.0001 |
| Post-Traumatic Stress Disorder | 83 (3.01) | 317 (2.94) | 0.8342 | 47 (2.00) | 302 (0.29) | <.0001 |
| Personality Disorders | 159 (5.77) | 406 (3.76) | <.0001 | 70 (2.99) | 904 (0.87) | <.0001 |
| Eating Disorders | 11 (0.4) | 71 (0.66) | 0.1184 | 11 (0.47) | 139 (0.13) | <.0001 |
| Autism Spectrum Disorder | 49 (1.78) | 122 (1.13) | 0.0065 | 27 (1.15) | 190 (0.18) | <.0001 |
| ADHD | 594 (21.56) | 1680 (15.57) | <.0001 | 411 (17.53) | 7048 (6.78) | <.0001 |
| Conduct Disorder | 74 (2.69) | 244 (2.26) | 0.1882 | 48 (2.05) | 426 (0.41) | <.0001 |
| Physical Diagnoses |  |  |  |  |  |  |
| Epilepsy | 4 (0.15) | 33 (0.31) | 0.1495 | 3 (0.13) | 307 (0.3) | 0.137 |
| Hypertension | 103 (3.74) | 523 (4.85) | 0.0135 | 82 (3.5) | 5527 (5.32) | <.0001 |
| Diabetes | 49 (1.78) | 249 (2.31) | 0.0914 | 42 (1.79) | 2573 (2.48) | 0.0343 |
| Congestive Heart Failure | 7 (0.25) | 22 (0.2) | 0.6105 | 6 (0.26) | 405 (0.39) | 0.3019 |
| Liver Disease | 80 (2.9) | 327 (3.03) | 0.7296 | 73 (3.11) | 2753 (2.65) | 0.1671 |
| Cancer | 24 (0.87) | 68 (0.63) | 0.169 | 28 (1.19) | 1384 (1.33) | 0.565 |
| Insomnia | 5 (0.18) | 4 (0.04) | 0.021 | 11 (0.47) | 217 (0.21) | 0.0071 |
| Charlson Comorbidity Index | 58 (2.11) | 223 (2.07) | 0.8977 | 53 (2.26) | 2406 (2.31) | 0.8614 |
| **Context** |  |  |  |  |  |  |
| Rurality |  |  | 0.0029 |  |  | <.0001 |
| Rural | 241 (8.75) | 1122 (10.4) |  | 234 (9.98) | 15978 (15.37) |  |
| Metropolitan Statistical Area | 2456 (89.15) | 9512 (88.13) |  | 2066 (88.1) | 85992 (82.74) |  |
| Unknown | 58 (2.11) | 159 (1.47) |  | 45 (1.92) | 1963 (1.89) |  |
| State of Residence - New Mexico | 639 (23.19) | 3377 (31.29) | <.0001 | 582 (24.82) | 25727 (24.75) | 0.9422 |
| Year |  |  | <.0001 |  |  | <.0001 |
| 2005 | 29 (1.05) | 194 (1.8) |  | 28 (1.19) | 1565 (1.51) |  |
| 2006 | 32 (1.16) | 181 (1.68) |  | 30 (1.28) | 2365 (2.28) |  |
| 2007 | 31 (1.13) | 148 (1.37) |  | 31 (1.32) | 2127 (2.05) |  |
| 2008 | 144 (5.23) | 725 (6.72) |  | 135 (5.76) | 6505 (6.26) |  |
| 2009 | 78 (2.83) | 415 (3.85) |  | 74 (3.16) | 4282 (4.12) |  |
| 2010 | 59 (2.14) | 638 (5.91) |  | 73 (3.11) | 4206 (4.05) |  |
| 2011 | 133 (4.83) | 827 (7.66) |  | 123 (5.25) | 5629 (5.42) |  |
| 2012 | 111 (4.03) | 668 (6.19) |  | 105 (4.48) | 4437 (4.27) |  |
| 2013 | 483 (17.53) | 1778 (16.47) |  | 386 (16.46) | 17012 (16.37) |  |
| 2014 | 371 (13.47) | 1299 (12.04) |  | 319 (13.6) | 13695 (13.18) |  |
| 2015 | 376 (13.65) | 1173 (10.87) |  | 324 (13.82) | 14597 (14.04) |  |
| 2016 | 375 (13.61) | 1157 (10.72) |  | 311 (13.26) | 12658 (12.18) |  |
| 2017 | 178 (6.46) | 549 (5.09) |  | 127 (5.42) | 5650 (5.44) |  |
| 2018 | 86 (3.12) | 254 (2.35) |  | 74 (3.16) | 2876 (2.77) |  |
| 2019 | 90 (3.27) | 270 (2.5) |  | 78 (3.33) | 2431 (2.34) |  |
| 2020 | 106 (3.85) | 309 (2.86) |  | 73 (3.11) | 2358 (2.27) |  |
| 2021 | 73 (2.65) | 208 (1.93) |  | 54 (2.3) | 1540 (1.48) |  |
| **Healthcare Utilization** |  |  |  |  |  |  |
| Psychotherapy |  |  | <.0001 |  |  | <.0001 |
| None | 1041 (37.79) | 6590 (61.06) |  | 1045 (44.56) | 99617 (95.85) |  |
| 1-4 Visits | 1264 (45.88) | 3092 (28.65) |  | 962 (41.02) | 2923 (2.81) |  |
| 5+ Visits | 450 (16.33) | 1111 (10.29) |  | 338 (14.41) | 1393 (1.34) |  |
| Any Psychiatric ED visits | 22 (0.8) | 121 (1.12) | 0.1392 | 8 (0.34) | 185 (0.18) | 0.0791 |
| Psychotropic Medications |  |  | <.0001 |  |  | <.0001 |
| Anticonvulsants | 276 (10.02) | 1373 (12.72) |  | 225 (9.59) | 12833 (12.35) |  |
| Antidepressants | 1291 (46.86) | 4731 (43.83) |  | 1147 (48.91) | 50034 (48.14) |  |
| Antipsychotics/Tranquilizers | 82 (2.98) | 601 (5.57) |  | 43 (1.83) | 1033 (0.99) |  |
| Anxiolytics/Sedatives/Hypnotics | 293 (10.64) | 1300 (12.04) |  | 264 (11.26) | 17662 (16.99) |  |
| Hypotensive Agents | 62 (2.25) | 224 (2.08) |  | 36 (1.54) | 1578 (1.52) |  |
| Stimulants | 751 (27.26) | 2564 (23.76) |  | 630 (26.87) | 20793 (20.01) |  |
| **Health Outcomes** |  |  |  |  |  |  |
| Medication Gap (30 Days) | 1457 (52.89) | 5140 (47.62) | <.0001 | 1226 (52.28) | 66520 (64) | <.0001 |
| Medication Gap (90 Days) | 1055 (38.29) | 3865 (35.81) | 0.0155 | 884 (37.7) | 51071 (49.14) | <.0001 |
| Medication Gap (1800 Days) | 660 (23.96) | 2437 (22.58) | 0.1245 | 554 (23.62) | 33913 (32.63) | <.0001 |
| Any Reduction in Days' Supply | 216 (7.84) | 1148 (10.64) | <.0001 | 187 (7.97) | 5740 (5.52) | <.0001 |
| Reduction in Days' Supply (> 7 days) | 179 (6.5) | 955 (8.85) | <.0001 | 157 (6.7) | 4861 (4.68) | <.0001 |
| Discontinuation of Prescribing | 1381 (50.13) | 5405 (50.08) | 0.9639 | 1148 (48.96) | 53364 (51.34) | 0.0221 |

**Figure A. COVARIATE BALANCE AFTER WEIGHTING**

***Psychiatry Cohort***


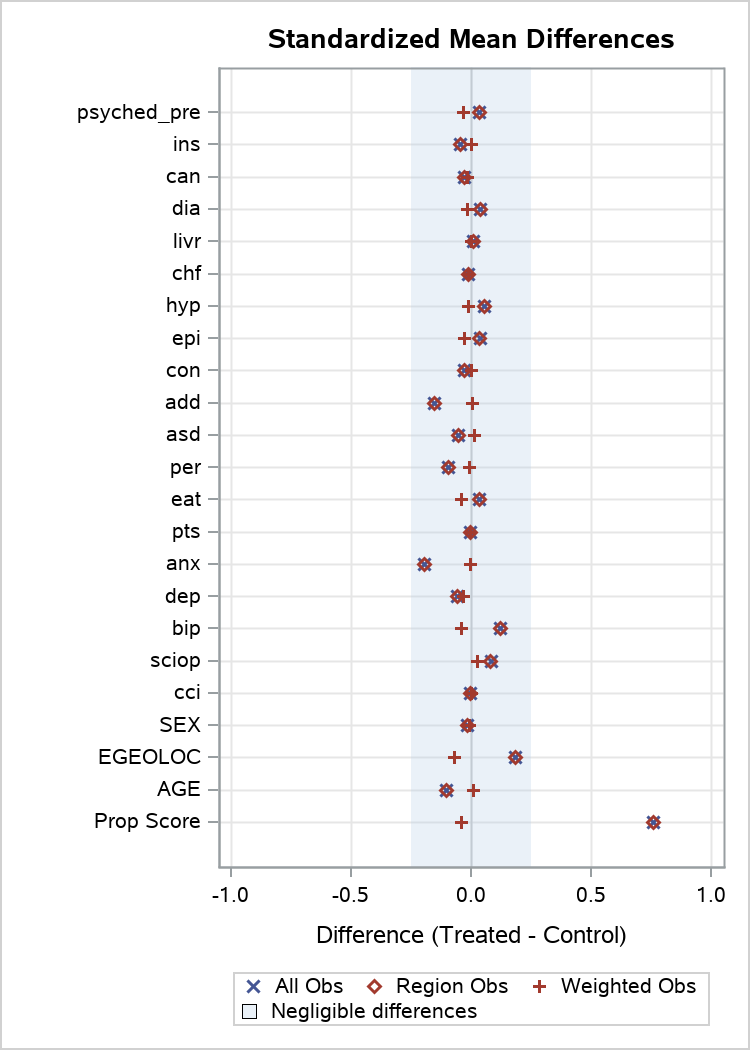


Note: psyched_pre = psychiatric emergency department utilization during 6-month lookback period; ins = Insomnia; can = cancer; dia = diabetes; livr = Liver disease; CHF = congestive heart failure; hyp = hypertension; epi = epilepsy; con = conduct disorder; add = attention deficit/hyperactivity disorder; asd = autism spectrum disorder; per = personality disorders; eat = eating disorders; pts = post-traumatic stress disorder; anx = anxiety disorders; dep = depressive disorders; bip = bipolar disorders; sciop = schizophrenia/other psychotic disorders; cci = Charlson Comorbidity Index score > 2; egeoloc = state of residence; prop score = propensity score.

| **Table B. Provider Type by year** | | | | | | | | | | | | | | | | | |
| --- | --- | --- | --- | --- | --- | --- | --- | --- | --- | --- | --- | --- | --- | --- | --- | --- | --- |
|  | **2005** | **2006** | **2007** | **2008** | **2009** | **2010** | **2011** | **2012** | **2013** | **2014** | **2015** | **2016** | **2017** | **2018** | **2019** | **2020** | **2021** |
|  | **%** | **%** | **%** | **%** | **%** | **%** | **%** | **%** | **%** | **%** | **%** | **%** | **%** | **%** | **%** | **%** | **%** |
| **Psychiatrist** | 1.66 | 1.58 | 1.34 | 6.45 | 3.65 | 5.16 | 7.13 | 5.77 | 16.60 | 12.31 | 11.42 | 11.22 | 5.35 | 2.50 | 2.71 | 3.08 | 2.08 |
| **Psychologist** | 1.86 | 1.82 | 1.71 | 7.03 | 3.88 | 5.93 | 8.32 | 6.06 | 14.95 | 11.54 | 10.97 | 10.46 | 5.02 | 2.43 | 3.01 | 3.11 | 1.90 |

| **Table C. Provider Type by Employment Status** | | | | |
| --- | --- | --- | --- | --- |
|  | **Employment Status** | | | |
|  | **Full-time** | **Part-time** | **Retired** | **Other / Unknown** |
|  | **%** | **%** | **%** | **%** |
| **Psychiatrist** | 29.95 | 0.52 | 1.07 | 68.46 |
| **Psychologist** | 31.03 | 0.69 | 0.97 | 67.31 |

| **Table D. Provider Type by Salary Type** | | | |
| --- | --- | --- | --- |
|  | **Salary Type** | | |
|  | **Salary** | **Hourly** | **Unknown** |
|  | **%** | **%** | **%** |
| **Psychiatrist** | 11.66 | 10.55 | 77.80 |
| **Psychologist** | 12.46 | 11.20 | 76.33 |

| **Table E. Provider Type by Relationship to Employee** | | | |
| --- | --- | --- | --- |
|  | **Relationship to Employee** | | |
|  | **Employee** | **Spouse** | **Child/Other** |
|  | **%** | **%** | **%** |
| **Psychiatrist** | 49.19 | 16.16 | 34.65 |
| **Psychologist** | 49.29 | 16.52 | 34.19 |

| **Table F. Provider Type by Rurality** | | | |
| --- | --- | --- | --- |
|  | **Rurality** | | |
|  | **Rural** | **Non-Rural** | **Unknown** |
|  | **%** | **%** | **%** |
| **Psychiatrist** | 10.01 | 88.38 | 1.61 |
| **Psychologist** | 9.01 | 89.51 | 1.48 |

| **Table G. Provider Type by Index Medication Type** | | | | | | |
| --- | --- | --- | --- | --- | --- | --- |
|  | **Medication Type** | | | | | |
|  | **Anticonvulsant** | **Antidepressant** | **Antipsychotic** | **Anxiolytic** | **Hypotensive** | **Stimulant** |
|  | **%** | **%** | **%** | **%** | **%** | **%** |
| **Psychiatrist** | 12.23 | 44.48 | 5.05 | 11.75 | 2.10 | 24.40 |
| **Psychologist** | 13.54 | 44.85 | 5.11 | 11.43 | 1.91 | 23.17 |

***Figure B. Primary Care Physician Cohort***


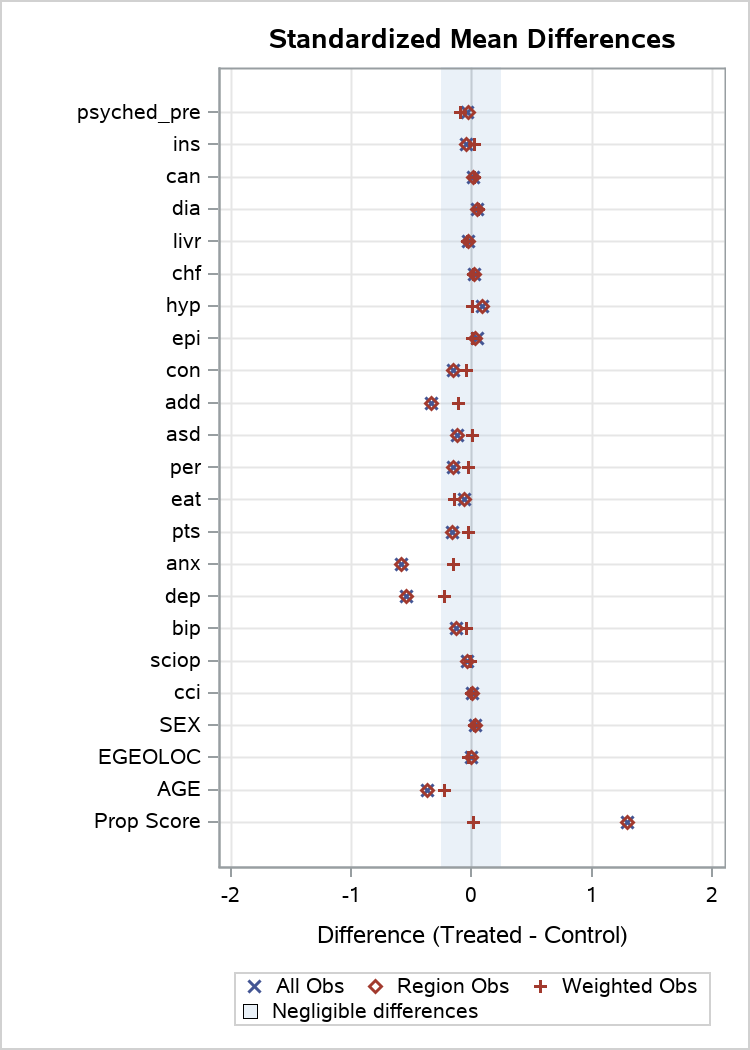


Note: psyched_pre = psychiatric emergency department utilization during 6-month lookback period; ins = Insomnia; can = cancer; dia = diabetes; livr = Liver disease; CHF = congestive heart failure; hyp = hypertension; epi = epilepsy; con = conduct disorder; add = attention deficit/hyperactivity disorder; asd = autism spectrum disorder; per = personality disorders; eat = eating disorders; pts = post-traumatic stress disorder; anx = anxiety disorders; dep = depressive disorders; bip = bipolar disorders; sciop = schizophrenia/other psychotic disorders; cci = Charlson Comorbidity Index score > 2; egeoloc = state of residence; prop score = propensity score.

| **Table H. Provider Type by year** | | | | | | | | | | | | | | | | | |
| --- | --- | --- | --- | --- | --- | --- | --- | --- | --- | --- | --- | --- | --- | --- | --- | --- | --- |
|  | **2005** | **2006** | **2007** | **2008** | **2009** | **2010** | **2011** | **2012** | **2013** | **2014** | **2015** | **2016** | **2017** | **2018** | **2019** | **2020** | **2021** |
|  | **%** | **%** | **%** | **%** | **%** | **%** | **%** | **%** | **%** | **%** | **%** | **%** | **%** | **%** | **%** | **%** | **%** |
| **Primary Care Physician** | 1.50 | 2.25 | 2.03 | 6.24 | 4.10 | 4.03 | 5.41 | 4.28 | 16.36 | 13.19 | 14.04 | 12.20 | 5.43 | 2.78 | 2.36 | 2.29 | 1.50 |
| **Psychologist** | 2.50 | 1.70 | 2.06 | 4.59 | 4.05 | 3.55 | 6.41 | 4.93 | 15.21 | 13.02 | 14.36 | 11.81 | 5.50 | 2.78 | 3.51 | 2.51 | 1.50 |

| **Table I. Provider Type by Employment Status** | | | | |
| --- | --- | --- | --- | --- |
|  | **Employment Status** | | | |
|  | **Full-time** | **Part-time** | **Retired** | **Other / Unknown** |
|  | **%** | **%** | **%** | **%** |
| **Primary Care Physician** | 27.94 | 0.47 | 0.96 | 70.63 |
| **Psychologist** | 33.45 | 0.25 | 0.60 | 65.70 |

| **Table J. Provider Type by Salary Type** | | | |
| --- | --- | --- | --- |
|  | **Salary Type** | | |
|  | **Salary** | **Hourly** | **Unknown** |
|  | **%** | **%** | **%** |
| **Primary Care Physician** | 9.77 | 10.94 | 79.29 |
| **Psychologist** | 11.43 | 14.08 | 74.49 |

| **Table K. Provider Type by Relationship to Employee** | | | |
| --- | --- | --- | --- |
|  | **Relationship to Employee** | | |
|  | **Employee** | **Spouse** | **Child/Other** |
|  | **%** | **%** | **%** |
| **Primary Care Physician** | 57.93 | 20.84 | 21.23 |
| **Psychologist** | 53.02 | 17.75 | 29.23 |

| **Table L. Provider Type by Rurality** | | | |
| --- | --- | --- | --- |
|  | **Rurality** | | |
|  | **Rural** | **Non-Rural** | **Unknown** |
|  | **%** | **%** | **%** |
| **Primary Care Physician** | 15.26 | 82.82 | 1.89 |
| **Psychologist** | 14.29 | 83.89 | 1.82 |

| **Table M. Provider Type by Index Medication Type** | | | | | | |
| --- | --- | --- | --- | --- | --- | --- |
|  | **Medication Type** | | | | | |
|  | **Anticonvulsant** | **Antidepressant** | **Antipsychotic** | **Anxiolytic** | **Hypotensive** | **Stimulant** |
|  | **%** | **%** | **%** | **%** | **%** | **%** |
| **Primary Care Physician** | 12.27 | 48.15 | 1.01 | 16.85 | 1.52 | 20.18 |
| **Psychologist** | 11.39 | 50.19 | 1.31 | 12.30 | 1.09 | 23.73 |
