# Supplementary material for: Psychotropic deprescribing across different prescribing professions in New Mexico and Louisiana
Source: PLOS Ment Health. 2025 Dec 30;2(12):e0000408. doi: 10.1371/journal.pmen.0000408 (PMC12798385; doi:10.1371/journal.pmen.0000408)
Supplement: S2 Text — Fig A. Days Discontinuation Without Replacement Among Patients of Prescribing Psychologists and Psychiatrists. Fig B. Days Discontinuation Without Replacement Among Patients of Prescribing Psychologists and Primary Care Physicians. Fig C. Days Until Complete Discontinuation of Prescribing Among Patients of Prescribing Psychologists and Psychiatrists. Fig D. Days Until Complete Discontinuation of Prescribing Among Patients of Prescribing Psychologists and Primary Care Physicians. Fig E. Days Until Sustained Reduction in Days’ Supply Among Patients of Prescribing Psychologists and Psychiatrists. Fig F. Days Until Sustained Reduction in Days’ Supply Among Patients of Prescribing Psychologists and Primary Care Physicians. (DOCX) [file pmen.0000408.s002.docx]

**S2 Text. Survival Curves**

**Contents:**

Fig A. Days Discontinuation Without Replacement Among Patients of Prescribing Psychologists and Psychiatrists.

Fig B. Days Discontinuation Without Replacement Among Patients of Prescribing Psychologists and Primary Care Physicians.

Fig C. Days Until Complete Discontinuation of Prescribing Among Patients of Prescribing Psychologists and Psychiatrists.

Fig D. Days Until Complete Discontinuation of Prescribing Among Patients of Prescribing Psychologists and Primary Care Physicians.

Fig E. Days Until Sustained Reduction in Days’ Supply Among Patients of Prescribing Psychologists and Psychiatrists

Fig F. Days Until Sustained Reduction in Days’ Supply Among Patients of Prescribing Psychologists and Primary Care Physicians.

**Figure A. Days Discontinuation Without Replacement Among Patients of Prescribing Psychologists and Psychiatrists.**


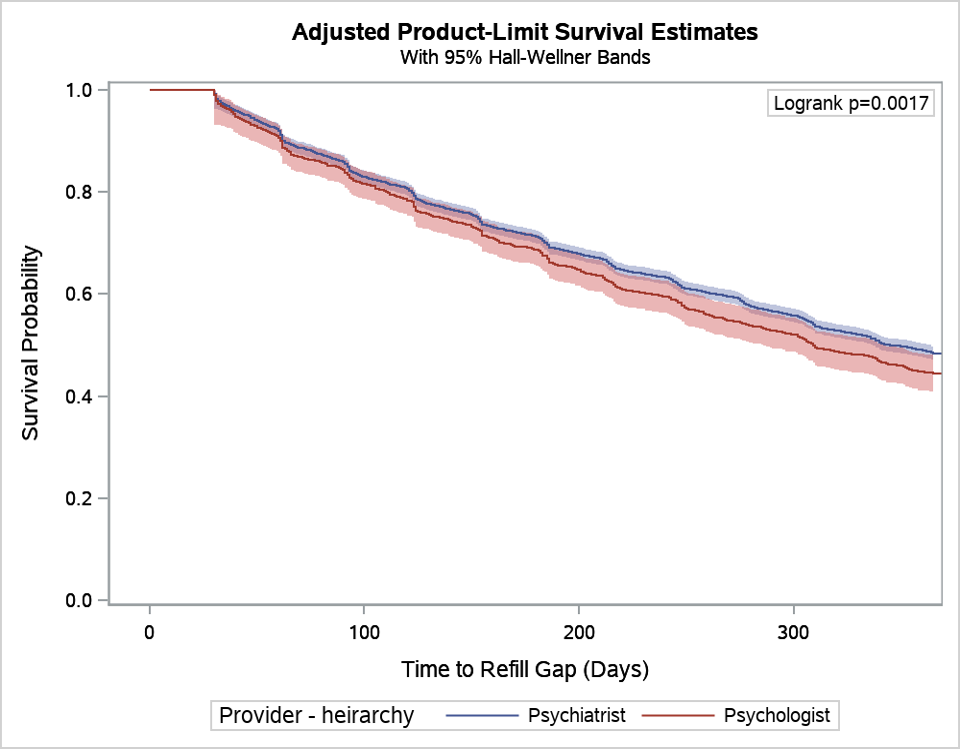


Note: Analysis is weighted using inverse propensity for treatment weighting (IPTW). Deprescribing was defined using at least a 30-day gap between the end of medication supply and the next prescription. As a result, patients could not experience deprescribing until after the end of day 30 of the study period. Censored observations not shown for clarity.

**Figure B. Days Discontinuation Without Replacement Among Patients of Prescribing Psychologists and Primary Care Physicians.**

**
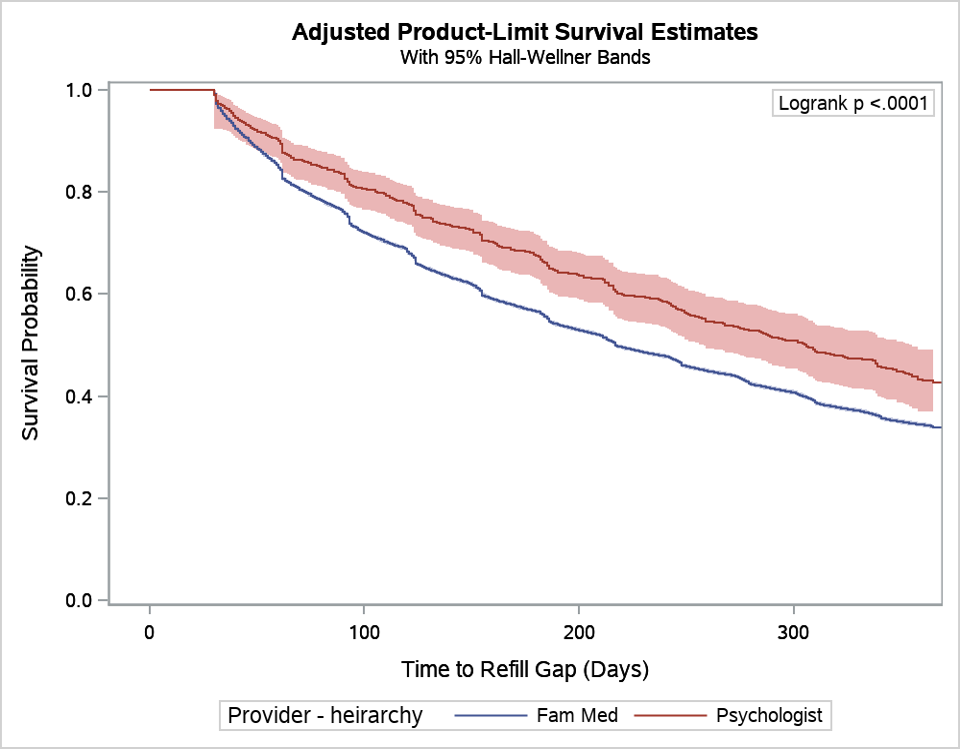
**

Note: “Fam Med” = Primary Care Physicians, including family medicine, internal medicine, and pediatrics. Analysis is weighted using inverse propensity for treatment weighting (IPTW). Deprescribing was defined using at least a 30-day gap between the end of medication supply and the next prescription. As a result, patients could not experience deprescribing until after the end of day 30 of the study period. Censored observations not shown for clarity.

**Figure C. Days Until Complete Discontinuation of Prescribing Among Patients of Prescribing Psychologists and Psychiatrists.**


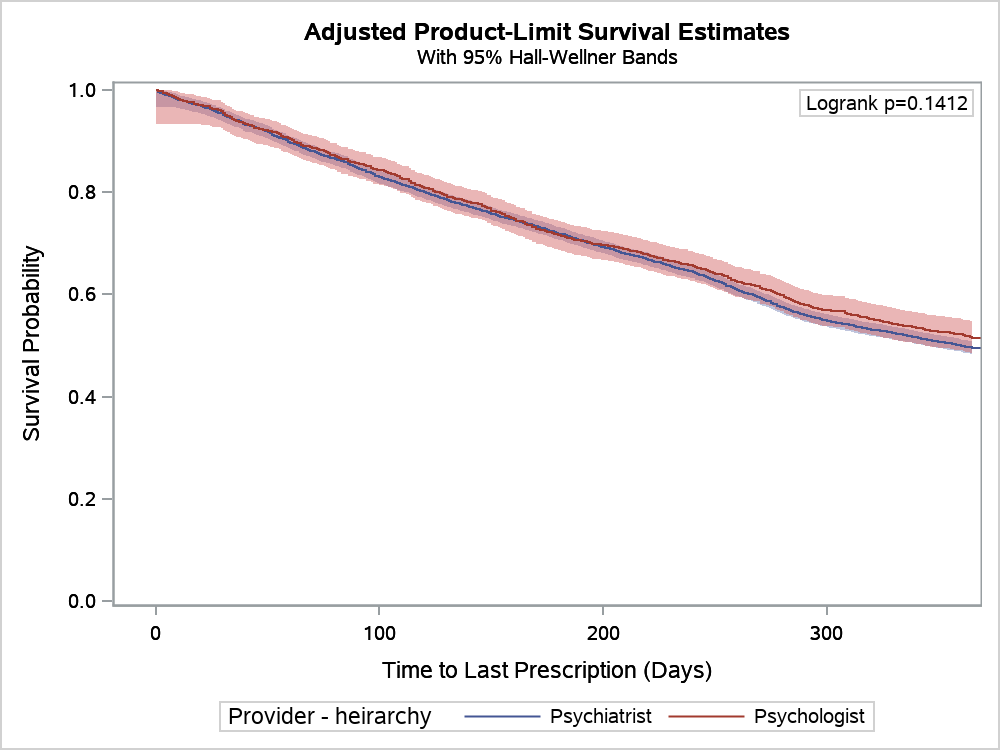


Note: Analysis is weighted using inverse propensity for treatment weighting (IPTW).

**Figure D. Days Until Complete Discontinuation of Prescribing Among Patients of Prescribing Psychologists and Primary Care Physicians.**


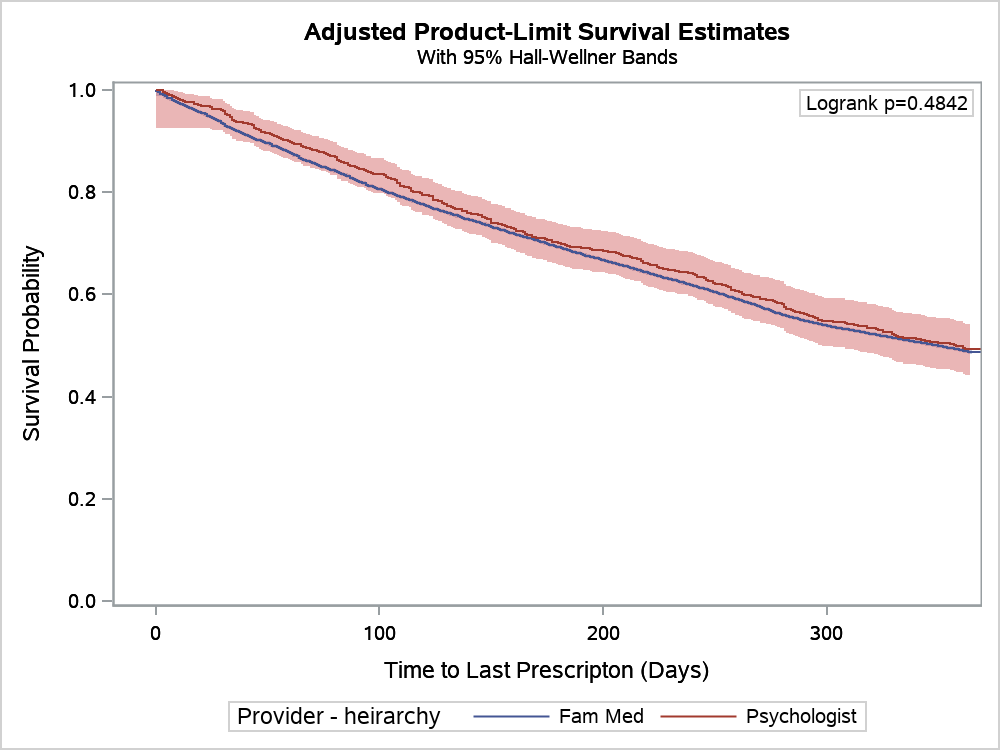


Note: Analysis is weighted using inverse propensity for treatment weighting (IPTW). Fam Med = Primary care physicians (including family medicine, internal medicine, and pediatrics).

**Figure E. Days Until Sustained Reduction in Days’ Supply Among Patients of Prescribing Psychologists and Psychiatrists.**


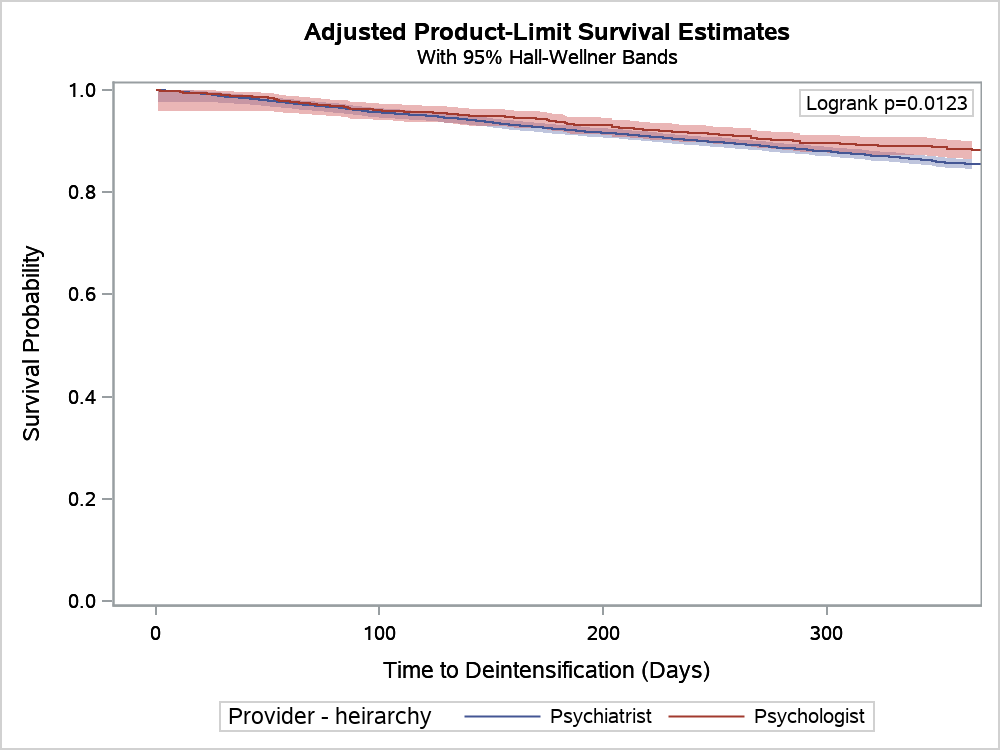


Note: Analysis is weighted using inverse propensity for treatment weighting (IPTW).

**Figure F. Days Until Sustained Reduction in Days’ Supply Among Patients of Prescribing Psychologists and Primary Care Physicians.**


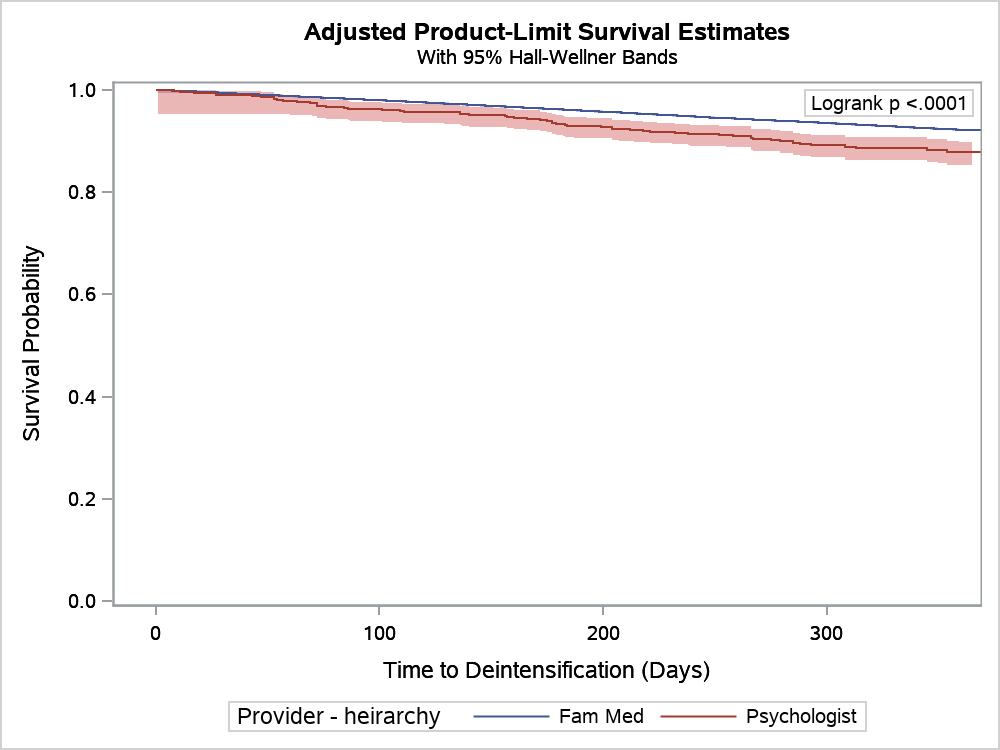


Note: Analysis is weighted using inverse propensity for treatment weighting (IPTW). Fam Med = Primary care physicians (including family medicine, internal medicine, and pediatrics).
