# Supplementary material for: Psychotropic deprescribing across different prescribing professions in New Mexico and Louisiana
Source: PLOS Ment Health. 2025 Dec 30;2(12):e0000408. doi: 10.1371/journal.pmen.0000408 (PMC12798385; doi:10.1371/journal.pmen.0000408)
Supplement: Attachment — Submitted filename: Response to Reviewer Comments.docx [file pmen.0000408.s004.docx]

**To the reviewers:**

Thank you for your thoughtful feedback on our manuscript “Psychotropic deprescribing across different prescribing professions in New Mexico and Louisiana.” Your comments were quite helpful in improving the overall readability of the manuscript, especially with respect to clarity in the introduction, a more balanced interpretation in the discussion, and identifying additional limitations worth noting. We made every effort to address each comment, and there were no comments with which we disagreed or provided a rebuttal.

We have included a point-by-point response to all comments below.

**Response to Reviewer Comments:**

| R1 | Abstract: 2 grammatical errors • line 28: “used to developed” should be “used to develop” • line 29: “from a psychologists, psychiatrists…” should be “from psychologists, psychiatrists…” | We have made these revisions |
| --- | --- | --- |
|  | Line 89-90: Please either revise this statement or add more context: “In addition to the potential for these deprescribing barriers and facilitators to disproportionately impact PCPs, the opposite may be true for prescribing psychologists.” It is unclear from the preceding paragraph and text following this sentence how would the opposite impact of any barriers or facilitators would be true for psychologists. Please add more information. | We have clarified this to state:  “In contrast to PCPs, who may encounter disproportionate barriers to deprescribing, psychologists are well-positioned to deprescribe due to their training and emphasis on the therapeutic relationship..”  Later in the paragraph, we have also added the following:  “Furthermore, given that the therapeutic alliance between patient and provider is a key component of psychotherapy, it is possible that prescribing psychologists fully benefit from the role of this relationship in facilitating successful deprescribing.” |
|  | Lines 105-106: This statement is presented as a given, but does not seem supported in the current text: “the potential for known deprescribing barriers to impact prescribers differently”. Please either revise or add text/citations to support this statement. | Thank you for highlighting this point of clarification, as we did not intend to convey this as a known fact but rather that this is an unknown but plausible scenario that further motivates the current study. We have changed this phrase to the following:  “the unexplored possibility for known deprescribing barriers to impact prescribers differently” |
|  | Lines 111-112: Suggest adding a citation to support the statement that long-term use of psychotropic medication could be warranted: “Finally, while deprescribing is considered a positive outcome in the context of this study, we also acknowledge that long-term medication usage may be the correct treatment decision for a given patient.” | We have added a citation and example. |
|  | Figures 1 & 2: Scanned quality of the figures is poor, ensure final submission includes higher quality graphics. | We included high-quality TIFF images for the figures – the poor image quality in the review copy appears to be a result of the editorial manager system compressing the image for review. |
|  | Line 157-158: Sentence needs revision to correct multiple grammatical errors: “Patients who do not experience deprescribing during the follow up were be considered censored at the end of the year or their last day of enrollment.” | We have made this revision. |
|  | Tables 1 and 2: The titles of both tables are identical “Hazard Ratios and 95% Confidence Intervals comparing Deprescribing Rates between Prescribing Psychologists and Psychiatrists” – I believe Table 2 refers to PCP, not Psychiatrists? | Thank you for catching this error. We have updated the heading for Table 2 to reflect the PCP cohort. |
|  | Line 317: Table 10 is referenced in error: “The doubly-robust model supported this (Table 10),…” | We have corrected this to Table 1 |
|  | Line 326-327: Table 11 is referenced in error: “The model supported this (Table 11),…” | We have corrected this to Table 2 |
|  | References: The authors are encouraged to format their references consistently according to the style of the journal – some of the cited titles have all words in upper case, and some only capitalize the first word of the journal title. Consistent formatting at the manuscript submission stage would reduce burden on the journal editing team. | We have reformatted the references to align with the Vancouver style. |
| R2 | (Line 165): How were inpatient services delineated for prescribing purposes. For example, if a patient was admitted to medicine with a psychiatry consult, was that patient then attributed to a PCP because the admitting attending was internal medicine? Clarification here would be helpful, because in these cases the subsequent PCP may be reluctant to deprescribe a medication started by a psychiatrist. | Thank you for this question. Given our use of insurance claims, patients can only be assigned to providers for whom they have billed visits. If the psychiatry consult resulted in the psychiatrist billing for services, the psychiatrist would be identified as the prescriber; if the psychiatrist did not bill independently, however, the PCP would be identified as the prescriber. We have made this reliance on billing more explicit:  “This approach examined the various providers with whom the patient had a billed visit in an outpatient or inpatient settings in the 30 days prior to the initial psychotropic prescription. If they ever had a billed visit for a psychiatrist, it was assumed that the prescription came from the psychiatrist.” |
|  | (Line 168): I would include some discussion here about how the possibility of a PCP starting a medication and simultaneously referring to psychiatry/psychology would be handled in this approach. Often wait times for specialist care may be quite long and this might lead claims to be attributed to PCPs that were actually managed by psychiatry/psychology. This would be particularly relevant if the the employer-based payers required PCP referral for specialist care rather than patients being able to access specialist care directly. | This is an excellent point, as this scenario could lead to misclassification. We have expanded the first limitation (measuring provider changes) to include limitations related to between-provider changes. We have also added the following in the methods to address this:  “For patients who initially received a prescription from a PCP but were subsequently referred to either a psychiatrist or prescribing psychologist, their prescription would only be attributed to the specialist provider if the specialist appointment was billed prior to the initial prescription fill. If the specialist appointment occurred after the initial prescription, then the PCP would be identified as the prescriber. Although this scenario is not unlikely, prior research with this cohort demonstrates that the PCP cohort is markedly different from the specialist cohorts, suggesting this may not be a significant problem.[23]” |
|  | There is no mention of how advanced practice providers are categorized. Where I practice, there is a high prevalence of both psychiatric and family medicine/internal medicine nurse practitioners. Additionally, the prevalence of physician assistance is quite high. If these prescribers are included in any one of the cohorts, this should be made very clear in the explanation of how claims are attributed. These advanced practice providers are neither psychiatrists nor family medicine/internal medicine/pediatric physicians and may have dramatically different prescribing practices. | We agree that APPs are highly valuable members of the care team and may have unique prescribing practices all their own that warrant examination. For the purposes of this study, we did not examine APPs. We have clarified this regarding our main prescriber type definition:  “Using this algorithm, visits with advanced practice providers (such as nurse practitioners and physician associates) were not examined in this study such that patients who did not see a psychiatrist, PCP, or psychologist were excluded.”  We have also included this as a limitation and future direction:  “the present study did not include advanced practice providers. Given the rapid growth of the psychiatric mental health nurse practitioner workforce,[41] there is a need for future research to examine the deprescribing practices of those providers relative to psychiatrists, PCPs, non-psychiatric nurse practitioners, and physician associates.” |
|  | Within the limitations section, I would question whether the use of private, employer-sponsored insurance is a limitation since the results may not be generalizable to the Medicare/Medicaid populations. | Thank you for this suggestion. We have added the following to the limitations:  “Third, there are aspects of our cohort selection that limit generalization. Given our use of private, employer-sponsored insurance claims, our results may not generalize well to publicly-insured patients covered by Medicaid or Medicare. This limitation would both reduce the representation of low-income and senior participants in the sample. In particular, a notably larger proportion of adults with mental illness are insured through Medicaid (33%) than private insurance (21%),[40] suggesting a need for future research to replicate this study using Medicaid claims.” |
|  | Within the limitations section, recommend adding a caveat about fragmented care. In my own practice, refills from PCPs seem to be perpetuated even when I've assumed care of the patient as their psychiatrist and so it is possible that claims data do not reflect what the clinician currently managing the patient's mental health care believe the patient is/should be taking. | This is an excellent point. We have expanded the first limitation (measuring provider changes) to include limitations related to fragmented care. |
|  | (Line 178): for the deprescribing sections, would clarify whether approach #3 (sustained reduction in prescribed days' supply) takes into account daily dose? In the section around line 178 it does not, but then line 361 refers to tapering, which typically takes a daily medication and systematically reduces the dose/day, rather than the days' supply. | Thank you for raising this question. We were unfortunately not able to assess dose reductions given the broad range of drugs contained within the classes examined, and we have now clarified this in the methods describing approach #3. In the discussion where tapering is mentioned as an example of a possible deintensification approach, we have expanded this to state “e.g., tapering via switching to a short course of lower dose medications or via pill splitting,” as these common types of tapering could be reflected in a reduced days’ supply. For example, a 28 day supply reduced to a 14 day supply may reflect 1) a 40mg pill being split to lower doses that would still cover the same 28 day period or 2) a switch from a 40mg long-term dose to a 2-week course of a lower dose as part of a taper. We have included a citation that documents pill splitting and the use of short intermediary prescriptions as common taper strategies. |
|  | (Line 349): question the assertion that high degree of deprescribing is a positive outcome of deprescribing. At least some of this may be related to patient/clinician unmet expectations of the benefits of psychotropic medications. Your assertion implies that the patient is doing better and therefore their clinician deprescribes, but a very different possibility remains that the patient is doing poorly on their medication and is tired of side effects so stops filling their prescriptions and continues to do poorly. Without outcomes data, this assertion is a stretch. | We agree that this is entirely possible and offers a more balanced interpretation. We have revised this section to state:  “While some patients may have conditions needing long-term management with psychotropic medications, the high degree of deprescribing occurring within the first year (after a 90-day initiation period) of starting a psychotropic medication can likely be viewed as a positive outcome may reflecting the growing emphasis on deprescribing among providers.[4–6] However, this may also reflect the adverse physical and psychological effects that contribute to many patients’ desire to discontinue these medications.[33] |
|  | (Lines 380-388): This section is also a stretch. While you conclude that additional research is needed, I think the universally positive hypotheses listed detract from the overall paper and may lead your readers to discount your research due to perceived bias. Would recommend restating as a broader "More research is necessary to elucidate the mechanisms behind the lower rate of deprescribing among psychologists"...or something along those lines. | We appreciate this feedback, as we had not initially interpreted these as universally positive (e.g., more cautious prescribing could also be overly cautious). We have revised this paragraph to the following:  “Additional research is needed to elucidate the mechanisms behind these deprescribing differences. Instead, based on these prior findings, we posit that the lower rate of deprescribing may reflect a more cautious approach to medication initiation used by prescribing psychologists as well as a tendency to pair medication with psychotherapy. We hypothesize that lower rates of psychotropic polypharmacy among patients of prescribing psychologists inherently reduces the number of medications from which an individual can be deprescribed, resulting in fewer opportunities to deprescribe rather than a lower proclivity for deprescribing. Additionally, prescribing psychologists may be starting patients on lower doses of psychotropic medication with the intention of using the medication as a supplement to psychotherapy rather than as the primary treatment modality. Future research is needed to examine this hypothesis. Alternatively, while prescribing psychologists are safe prescribers, this may indicate that deprescribing remains an area of expertise in which psychiatrists remain uniquely skilled. Further complicating the interpretation of this relationship is that our use of insurance claims precludes us from assessing the reason for deprescribing. It is possible that different provider types are more adept at identifying when a medication is no longer needed, while others may be more attuned to patient feedback regarding symptoms, and those different rationales for deprescribing may lead to different deprescribing approaches. Differences in deprescribing may also reflect resource variation within their respective clinical settings. For example, PCPs practicing in interdisciplinary primary care practices may have access to clinical pharmacists who can highlight deprescribing opportunities, while psychiatrists and prescribing psychologists in private practice may not. Qualitative interviews with prescribing psychologists, psychiatrists, and PCPs regarding their deprescribing experiences may shed light on the unique relationships between each provider type and deprescribing. |
